# Peripheral Blood MicroRNAs as Potential Biomarkers of Myocardial Damage in Acute Viral Myocarditis

**DOI:** 10.3390/genes12030420

**Published:** 2021-03-15

**Authors:** Maria Marketou, Joanna Kontaraki, Alexandros Patrianakos, George Kochiadakis, Ioannis Anastasiou, Konstantinos Fragkiadakis, Anthoula Plevritaki, Sofia Thalia Papadaki, Gregory Chlouverakis, Fragiskos Parthenakis

**Affiliations:** 1 Cardiology Department, University General Hospital of Heraklion, 71110 Heraklion, Crete, Greece; apatrianakos@yahoo.gr (A.P.); kochiad@med.uoc.gr (G.K.); ioannisaanastassiou@yahoo.gr (I.A.); fragkiadakisk@hotmail.com (K.F.); anthiplevritaki@gmail.com (A.P.); sofia.thalia.papadaki@hotmail.com (S.T.P.); parthenf@uoc.gr (F.P.); 2 Department of Molecular Cardiology, School of Medicine, University of Crete, 71110 Heraklion, Crete, Greece; kontarak@med.uoc.gr; 3 Division of Biostatistics, School of Medicine, University of Crete, 71110 Heraklion, Crete, Greece; gchlouve@uoc.gr

**Keywords:** myocarditis, microRNA, troponine, strain

## Abstract

Background: microRNAs (miRs) have emerged as important modulators of cardiovascular development and disease. Our aim was to determine whether cardiac-related miRs such as miR-21-5p and miR-1-3p were differentially expressed in acute viral myocarditis and whether any of them was related with the extent of myocardial damage and left ventricular dysfunction. Methods: We enrolled 40 patients with acute viral myocarditis. Blood samples were taken on admission and miRs expression levels in peripheral blood mononuclear cells were quantified by real-time reverse transcription polymerase chain reaction. Results: miR-21-5p, miR-1-3p were significantly elevated in acute myocarditis. miR-21-5p levels showed a strong correlation with global longitudinal strain (*r* = 0.71, *p* < 0.01), while miR-1-3p had significant correlations with troponin I (*r* = 0.79, *p* < 0.01). Conclusions: The expression of miR-21-5p and miR-1-3p in peripheral blood is increased in acute viral myocarditis, and this increase is correlated with myocardial damage and indicative of left ventricular systolic dysfunction in these patients.

## 1. Introduction

Acute myocarditis is characterized by inflammatory myocardial damage and is associated with considerable morbidity and mortality, not only in the acute phase but also in the long term [1]. The pathophysiology of myocarditis is complex, its diagnosis may be challenging, while its clinical course is often unpredictable, highly variable and poorly understood [1].

A significant proportion of patients with acute or chronic myocarditis have preserved left ventricular ejection fraction, with no indication of a functional deficit on routine echocardiographic measurements [1]. For this reason, it would be of great interest to find markers associated with its clinical manifestations or, even better, with the magnitude of the subclinical damage it causes. The prompt diagnosis of acute myocarditis presents a challenge, as does the evaluation of these patients’ entire clinical and laboratory profile, insofar as it may affect their long-term outcomes.

Newer ultrasound techniques, specifically 2D speckle-tracking echocardiography, can highlight subclinical left ventricular dysfunction in patients with myocarditis and preserved left ventricular function, even those without regional wall motion abnormalities [2]. Although left ventricular ejection fraction is a powerful predictor of mortality and is used for guiding treatment decisions, left ventricular global longitudinal strain (GLS) may be an independent predictor of functional recovery during acute follow-up [3] and may have an incremental independent prognostic value [4]. Many patients with a history of myocarditis continue to present in the long term a subclinical left ventricular dysfunction that is detectable by speckle-tracking echocardiography, even though left ventricular ejection fraction is conserved [5].

MicroRNAs (miRs) are small non-coding RNAs that play a critical role in the post-transcriptional regulation of protein expression by binding messenger RNAs [6]. There is evidence suggesting that miRs in peripheral blood may have a significant diagnostic potential [7,8]. miRs play important roles in the onset and development of a viral infection, while altered miRs expression is a response to this [9,10]. Changes in the expression of miRs, such as miR-21-5p and miR-1-3p are implicated in the pathogenesis and adverse outcome of viral myocarditis as several experimental and clinical studies [11,12,13]. These miRs have been introduced as biomarkers for different cardiac pathologic conditions and have been listed among others as cardio-specific miRs or cardio-miRs [14]. They are involved in the pathophysiology of several cardiovascular diseases and seem to be correlated with plasma troponin in acute coronary syndromes, which is one of the strongest biomarkers of cardiomyocyte damage [14,15,16].

In the present study, we sought to determine whether the cardiac-related miR-21-5p and miR-1-3p were differentially expressed in the peripheral blood mononuclear cells of patients with acute viral myocarditis. We have investigated these miRs in peripheral blood mononuclear cells (PBMCs) since the importance of transcriptomic and proteomic profile changes in those cells has been indicated in several cardiovascular conditions [17,18]. In addition, these cells have a critical pathophysiologic role in the immune response and the inflammatory reactions [19]. We also investigated whether any of these were associated with the extent of heart damage, as well as left ventricular dysfunction expressed by GLS, an index of myocardial dysfunction [20] that has previously been shown to be more sensitive than left ventricular ejection fraction [21].

## 2. Methods

### 2.1. Study Population

This was a single-center prospective study. From January to December 2017, we screened consecutive patients admitted to our department for acute myocarditis. Myocarditis was diagnosed based on recent symptomatology (<10 days), clinical presentation, elevation of troponin levels, electrocardiogram (ECG) and cardiac magnetic resonance (CMR) imaging [22]. All patients had to have had a febrile infection during the last 3 months before admission. Symptom onset was defined by the occurrence of at least one of the following complaints: chest pain, dyspnea, new-onset or worsening heart failure, severe arrhythmias or syncope. A detailed history, together with clinical, laboratory and echo data, were thoroughly analyzed and the appropriateness of the diagnosis was reviewed in every case.

We excluded patients with coronary artery disease, myositis, or renal failure. Other exclusion criteria were as follows: left ventricular ejection fraction <40% on admission, previously diagnosed cardiomyopathy or a family history of cardiomyopathy; a history of using potentially cardiotoxic drugs; autoimmune disease; cardiogenic shock, congenital heart disease, dilated or hypertrophic cardiomyopathy, and arrhythmias not related to myocarditis; or other immune diseases; and treatment with glucocorticoids or immunosuppressants before specimen collection. In all patients, obstructive coronary artery disease was excluded through coronary angiography, either by cardiac catheterization or by computed tomography if the pretest probability for coronary artery disease was very low [22].

Control patients were healthy volunteers, age- and sex-matched with the myocarditis patients, who visited the emergency department for atypical symptoms (nausea, dizziness, pricking thoracic chest pain or upper back pain, etc.) and were found to have a healthy cardiovascular system.

The study was carried out in accordance with the ethical guidelines of the Declaration of Helsinki of 1975, and the study protocol was approved by our hospital’s Scientific and Ethics Committee and by the hospital administration (study protocol number 3993, 1/10/12). All participants signed an informed consent document.

### 2.2. Echocardiographic Study

A standard M-mode and 2-dimensional (2D) echocardiographic study was performed in all participants, in accordance with the recommendations of the American Society of Echocardiography and the European Association of Echocardiography [23,24]. The study in patients with acute myocarditis was performed during the first 48 h of their admission. The 2D speckle-tracking strain analyses were performed on grayscale images of the left ventricle, using Echopac (GE Medical Systems, Chicago, Illinois, United States), and peak GLS was measured.

During strain analysis, the endocardial border was manually traced at end-systole and the width of the region of interest was manually adjusted to include the entire myocardial wall thickness. The Echopac software then automatically tracks and accepts segments with good tracking quality and rejects poorly tracked segments. However, the operator is able to manually override computer-generated tracking and accept or reject individual segments based on a visual assessment of the tracking quality.

All GLS measurements were performed by two experienced investigators blindly. The intra-observer variability in GLS measurements was <5%.

### 2.3. Cardiac Magnetic Resonance

All patients with myocarditis underwent CMR imaging with late gadolinium enhancement during the first week after their admission to the hospital. The test was performed according to the Lake-Louise criteria of the International Consensus Group on CMR Diagnosis of Myocarditis [25]. Intravenous injection of gadolinium diethylenetriaminepentaacetate was used to determine late enhancement.

### 2.4. RNA Isolation and miRs Quantification

Blood samples were taken from all patients on the day of admission. Peripheral blood mononuclear cells were isolated from 3 mL blood samples by density gradient centrifugation using Lymphoprep (Stem Cell Technologies Inc., Vancouver, BC, Canada) resuspended in TRI-Reagent (Ambion, Life Technologies, Carlsbad, CA, USA) and stored at −80 °C Total RNA was isolated and 1μg was reverse-transcribed using the MiR-X miRNA First-Strand Synthesis kit (Clontech, Takara Bio Inc., Otsu, Shiga, Japan). Measurements of miRs levels were performed by quantitative real-time polymerase chain reaction (qPCR) running for 40 cycles, using the Corbett Research 6000 detection system. The KAPA SYBR FAST qPCR Kit (Kapa Biosystems, Woburn, MA, USA) was used for qPCR assays. Primers used were 5′-TGG AAT GTA AAG AAG TAT GTA T-3′ for miR-1-3p, and 5′-TAG CTT ATC AGA CTG ATG TTG A-3′ for miR-21-5p. The standard curve method was used for absolute quantification of the amplification products and specificity was determined by performing a melting curve analysis. U6 expression was used as a normalization standard as suggested by the MiR-X miRNA First-Strand Synthesis kit (Clontech, Takara Bio Inc., Otsu, Shiga, Japan) using the U6 Forward Primer and the U6 Reverse Primer provided by the kit. Relative quantification of the amplification products was performed using the comparative delta-delta Ct (2^−ddCt^) method. All samples were run in duplicate and Ct values were averaged for the replicates.

### 2.5. Statistical Analysis

Summary descriptive statistics are presented as mean ± standard deviation or frequency (%), as appropriate. Comparisons of continuous variables between myocarditis and normal groups were performed using independent samples t-tests, or Mann–Whitney tests, as appropriate. Scatterplots and Pearson’s correlation coefficient were used to assess the association between continuous variates. All statistical tests were performed at the two-sided level of α = 5%. IBM-SPSS 25 software was used for all analyses.

## 3. Results

Our study included 40 patients with acute myocarditis (32 men, average age for both males and females: 24 ± 7 years) who were compared with a group of 29 healthy age- and sex-matched individuals (23 men, average age for both males and females: 23 ± 6 years). The mean interval from the onset of symptoms of the viral infection to the clinical presentation of myocarditis was 3.2 ± 2.4 months. Classic diffuse ST-segment abnormalities on the ECG were observed in 21 patients. Ten patients underwent invasive coronary angiography. None of the patients had coronary artery disease. Baseline characteristics and echocardiographic parameters of both groups are shown in Table 1. Late gadolinium enhancement was found in all patients and was observed in all walls that had partial motion disorders on echocardiography.

As expected, patients with acute myocarditis had significantly higher inflammation markers (C-reactive protein) and white blood cell counts than the control group, as well as significantly higher levels of troponin I and brain natriuretic peptide (BNP). Overall, not only left ventricular ejection fraction but also GLS was significantly lower (−16.9 ± 3%) in patients with myocarditis (Table 1) compared to age-controlled normal subjects (*p* < 0.001).

**Figure 1:**
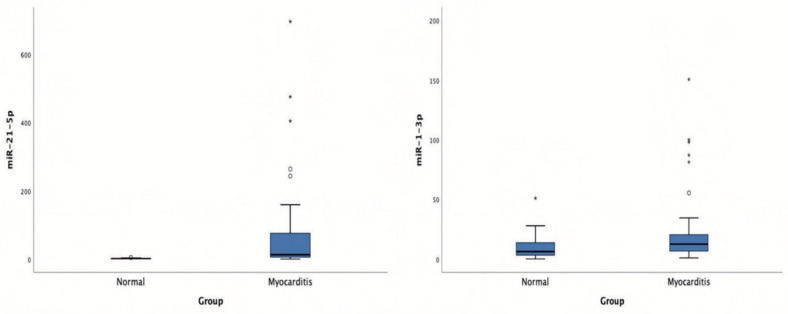
Boxplots of microRNA-21-5p (miR-21-5p) and microRNA-1-3p (miR-1-3p) expression levels in patients with acute viral myocarditis and in the control group.

**Figure 2:**
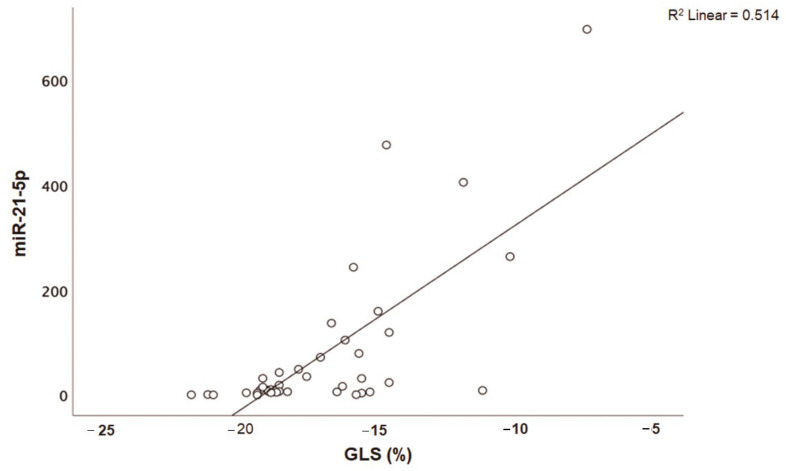
Correlation between global longitudinal strain (GLS, %) and microRNA-21-5p (miR-21-5p) expression levels (arbitrary units calculated by the (2^−ddCt^) method) in patients with acute myocarditis (*r* = 0.71, *p* < 0.01).

**Figure 3:**
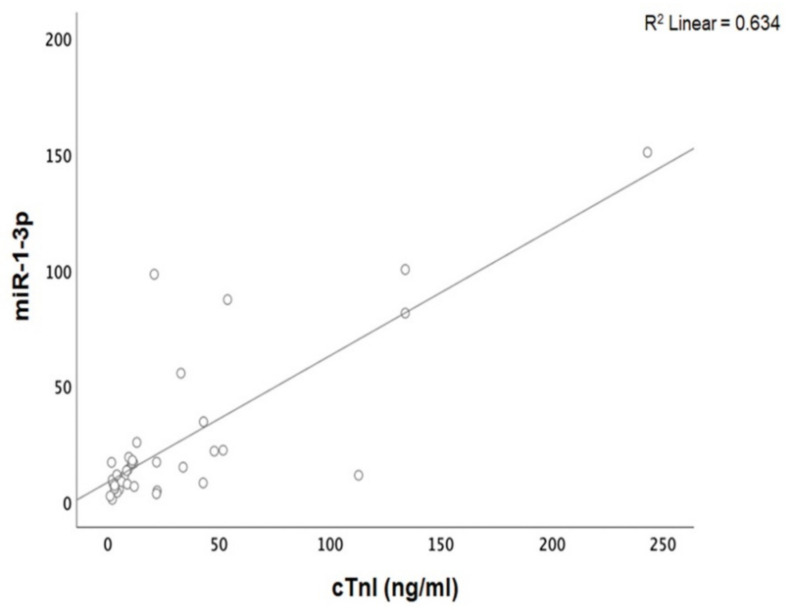
Correlation between cardiac troponin I (cTnI) levels and microRNA-1-3p (miR-1-3p) expression levels (arbitrary units calculated by the (2^−ddCt^) method) levels in patients with acute myocarditis: (*r* = 0.79, *p* < 0.01).

**Figure 4:**
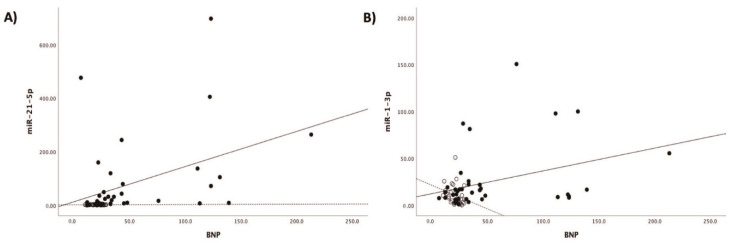
Correlation between (**A**) brain natriuretic peptide levels (BNP) and microRNA-1-3p (miR-1-3p) (*r* = 0.35, *p* = 0.03) and (**B**) brain natriuretic peptide levels (BNP) and microRNA-21-5p (miR-21-5p) (*r* = 0.41, *p* = 0.008) expression levels (arbitrary units calculated by the (2^−ddCt^) method) in patients with acute myocarditis.

Patients with acute myocarditis presented a distinct profile regarding the expression of certain miRs (Figure 1). More specifically, they showed significantly higher levels of both miRs that we studied, including miR-21-5p and miR-1-3p compared to their respective levels in the control group (Table 1, Table S1). Finally, correlating with indicators that directly or indirectly indicate acute myocardial damage in myocarditis, we found that there was a strong correlation between miR-21-5p levels and GLS in patients with acute myocarditis (*r* = 0.71, *p* < 0.01; Figure 2). Specifically, as GLS deteriorated and left ventricular systolic function worsened, miR-21-5p levels were elevated in most patients (Figure 1). On the other hand, there were significant correlations between the extent of myocardial damage, as expressed by troponin I and miR-1-3p (*r* = 0.79, *p* < 0.01, Figure 3). Finally, moderate correlations were observed between brain natriuretic peptide levels and miR-21-5p and miR-1-3p (*r* = 0.41, *p* = 0.008, *r* = 0.35, *p* = 0.03, respectively) (Figure 4).

## 4. Discussion

Our findings lead us to the conclusion that patients with acute viral myocarditis have a distinct profile in the expression of miRs such as miR-21-5p and miR-1-3p. Among these, miR-21-5p is strongly associated with left ventricular systolic dysfunction as expressed by GLS, while miR-1-3p is associated with the extent of myocardial damage and specifically with troponin I levels.

miRs have emerged as a class of endogenous, small, noncoding RNAs that regulate the gene expression of their target miRs. Tissue miRs can be released into circulating blood; they also offer new perspectives for developing biomarkers of cardiac diseases [9,10]. The literature has dealt extensively with the role of miRs in cardiovascular disease, where their expression seems to be decisive. Strong evidence indicates a diagnostic and therapeutic potential for miRs in several cardiovascular diseases. There are recent studies on the role of some miRs in inflammatory disease of the myocardium and myocarditis; however, data from human studies are scanty. miRs dysregulation is a characteristic of both human and mouse viral myocarditis [26,27]. The involvement of miRs and their usefulness as therapeutic targets in this process are not well studied. We know that miR-21-5p is consistently and strongly upregulated during acute myocarditis in both humans and susceptible mice [28].

It should be noted that the modulation of miR-21-5p in heart regulates the expression of matrix metalloprotease-2, which is an important factor in the pathophysiology of myocardial fibrosis and consequently in left ventricular remodeling and function [29]. A previous study in a pediatric population has indicated the importance of serum circulating miR-21-5p as a potential biomarker in myocarditis [30]. On the other hand, miR-1-3p is known to be involved in the pathophysiology of viral myocarditis via post-transcriptional repression of connexin 43, as indicated in myocardial cells in an experimental study of a myocarditis mouse model [31].

Speckle-tracking echocardiography is a useful method for detecting early myocardial dysfunction and evaluating treatment outcomes in acute viral myocarditis. GLS is a direct echocardiographic assessment of myocardial fiber deformation [32] that has been previously shown to be a more sensitive measurement of myocardial dysfunction than left ventricular ejection fraction [30]. It has also been shown to be a more powerful predictor of outcomes in patients who have heart failure with a reduced ejection fraction [21]. GLS appears to be impaired in up to 15% of patients with acute myocarditis, even those with a preserved ejection fraction, and GLS impairment in myocarditis (especially in myocardium) may persist during a two-year follow-up period, despite a preserved left ventricular ejection fraction [33,34]. Our study is the first to investigate the expression of miRs in connection with subclinical disorders of left ventricular systolic function in patients suffering from myocarditis, as expressed by left ventricular strain.

Our findings are not consistent with the entire literature, since there are conflicting results and published studies are not always in agreement. A previous experimental study suggests that myocardial miR-21-5p expression may be negatively related to the severity of viral myocarditis [35]. However, miRs expression and control mechanisms are complex and depend on many factors that often differ between studies and are always difficult to interpret. These conflicting results may arise from the fact that different sources of miRNAs have been analyzed in different studies. We have examined miRs expression in peripheral blood mononuclear cells which might not be in accordance with the corresponding expression in heart or in serum. In addition, we cannot exclude the possibility that the use of different normalizers and different Rt-qPCR methods, may be likely sources of variability among different studies. Nevertheless, it is clear that these miRs play regulatory roles in the pathophysiological mechanisms of acute myocarditis and are likely to have diagnostic potential in the detection of patients with subclinical damage.

We cannot provide a definite biologic explanation for our findings. We hypothesize that alterations of these miRs in PBMCs may represent the inflammatory burden of the disease that has an adverse impact on myocardial function and damage. In addition, we know that miRs participate in intercellular communication, are released from most cells, and disseminate through the extracellular fluid to reach other target cells, that in this case might be myocardial cells [36].

We examined miRs in PBMCs. Apart for the pathophysiologic relation of these cells with inflammatory conditions such as the myocarditis, the detection of miRs in serum or plasma has two major disadvantages. The first disadvantage is that their tissue of origin is not known. The second disadvantage is that no standardized method for the isolation and quantification of circulating microRNAs has been established so far and the miRs detection in serum is not that accurate (i.e., an accurate endogenous control, such as U6) for normalization of the qPCR reactions.

The diagnosis and clinical evaluation of myocarditis is challenging, and there is a lack of an effective approach to myocarditis management that mandates a deeper understanding of the basic molecular mechanisms of its pathophysiology. The majority of information about the pathophysiology of myocarditis has been derived from experimental studies, so data from relevant human studies are valuable for enriching our understanding of the disease. Notably, the prompt diagnosis of acute myocarditis and the clinical profile of these patients present a challenge. We focused on the evaluation of distinct profiles of miRs expression in acute myocarditis and more importantly to the relation between them and the extent of myocardial damage and left ventricular dysfunction. Our results indicate a possible pathophysiologic link between miR-1 and mir-21 in PBMCs in acute myocarditis and possible biomarkers that may contribute to a better understanding of the state of the disease and the clinical profile of these patients. Although they may add as biomarkers to the acute myocarditis monitor and management, studies are needed to further characterize this marker in patients with acute myocarditis and to distinguish it from the other biochemical markers of myocyte injury.

### Limitations

Endomyocardial biopsy was not performed in any patient. Although it has been considered as the gold standard for establishing the diagnosis of myocarditis, the significant limitations of the method have limited its implementation in usual clinical practice to only a few specific indications, while in other cases this approach is unnecessary or even contraindicated.

The sample size was small, and the study reflects a single center experience, based on a series of selected consecutive patients. However, our findings clearly allow us to reach definite clinical conclusions.

We also do not have a long-term follow-up of these patients that would allow us to assess whether our data have prognostic value and whether there is any correlation with the patients’ clinical outcomes. This should be the purpose of another larger and differently designed study, where further investigation will address the impact of this finding in the long term.

## 5. Conclusions

Our study demonstrates an association between cardiac damage and the expression of miR-21-5p and miR-1-3p in the pathological process of acute myocarditis. miR-21-5p is strongly associated with left ventricular systolic dysfunction while miR-1-3p is increased with the extent of myocardial damage. Peripheral blood miR-21-5p and miR-1-3p may serve as surrogate markers in the clinical evaluation of patients with acute viral myocarditis. Our study reinforces the hypothesis that, since they have a regulatory pathophysiological role in myocarditis, miRs could also become therapeutic targets, providing novel approaches to this potentially devastating disease.

## Figures and Tables

**Table 1:** Demographic and clinical characteristics of enrolled participants.

	Acute Myocarditis (*n* = 40)	Healthy Individuals (*n* = 29)	95% Confidence Interval of the Difference	*p*
Age (years)	24 ± 7	23 ± 6	−1.5–4.5	0.32
BMI (kg/m^2^)	26.5 ± 3.7	25.7 ± 3.2	−0.1–1.5	0.65
SBP (mmHg)	111 ± 22	112 ± 24	−2.5–2.1	0.43
DBP (mmHg)	70 ± 8	72 ± 10	−1.5–2.5	0.48
Heart rate (bpm)	77 ± 7	67 ± 6	7.8–13.7	<0.001
Hct (%)	41.6 ± 3.5	40.1 ± 2.5	−0.9–1.9	0.49
Creatinine (mg/dL)	0.96 ± 0.2	0.98 ± 0.1	−0.08–0.2	0.34
Troponine I (pg/mL)	29.5 ± 48	1.6 ± 0.2	11.4–46.3	<0.001
Brain natriuretic peptide (pg/mL)	50 ± 48	20 ± 6	10.5–18.5	<0.001
CRP (ng/mL)	5.5 ± 4	0.7 ± 0.3	3.1–5.5	<0.001
WBC (10^3^)	13,670 ± 2910	6890 ± 1180	5635–7927	<0.001
microRNA-21-5p (arbitrary units calculated by the (2^−ddCt^) method)	78.2 ± 147.9	1.8 ± 1	21.5–131.2	0.007
microRNA-1-3p (arbitrary units calculated by the (2^−ddCt^) method)	24.4 ± 32.9	10.8 ± 11.1	0.9–26.3	0.03
LVEF (%)	55 ± 10	59 ± 4	−0.8–0.5	0.027
LVEDD (mm)	48 ± 4	46 ± 5	−0.4–3.4	0.12
Left atrial volume index (mL/m^2^)	27 ± 6	26 ± 7	−0.2–1.1	0.24
GLS (%)	−16.9 ± 3	−19 ± 0.9	1–3.2	0.001
Patient with kinetic disorders in echocardiogram	36	0		
Hypokinesis of apical interventricular septum	25	-		
Hypokinesis of inferolateral wall	16	-		
Hypokinesis of apex	10	-		
Hypokinesis of lateral wall	11	-		
Hypokinesis of inferior wall	13	-		

Values are expressed as mean ± SD. BMI: body mass index; CRP: C-reactive protein; DBP: diastolic blood pressure; Hct: hematocrit; GLS: global longitudinal strain; LVEDD: left ventricular end diastolic diameter; LVEF: left ventricular ejection fraction; SBP: systolic blood pressure; WBC: white blood cells.

## Data Availability

The data that support the findings of this study are available from the corresponding author, [M.M.], upon reasonable request.

## Supplementary Materials

The following are available online at https://www.mdpi.com/2073-4425/12/3/420/s1, Table S1: Ct values and 2^−ddCt^ calculations for all the patients and the normal healthy donors.
